# Long-term morphological and hormonal follow-up in a single unit on 115 patients with adrenal incidentalomas

**DOI:** 10.1038/sj.bjc.6602459

**Published:** 2005-03-15

**Authors:** G P Bernini, A Moretti, C Oriandini, M Bardini, C Taurino, A Salvetti

**Affiliations:** 1 Department of Internal Medicine, University of Pisa, Via Roma 67, 56100 Pisa, Italy; 2 Department of Oncology, University of Pisa, Pisa, Italy

**Keywords:** adrenal incidentalomas

## Abstract

We investigated the natural course of adrenal incidentalomas in 115 patients by means of a long-term endocrine and morphological (CT) follow-up protocol (median 4 year, range 1–7 year). At entry, we observed 61 subclinical hormonal alterations in 43 patients (mainly concerning the ACTH–cortisol axis), but confirmatory tests always excluded specific endocrine diseases. In all cases radiologic signs of benignity were present. Mean values of the hormones examined at last follow-up did not differ from those recorded at entry. However in individual patients several variations were observed. In particular, 57 endocrine alterations found in 43 patients (37.2%) were no longer confirmed at follow-up, while 35 new alterations in 31 patients (26.9%) appeared *de novo*. Only four alterations in three patients (2.6%) persisted. Confirmatory tests were always negative for specific endocrine diseases. No variation in mean mass size was found between values at entry (25.4±0.9 mm) and at follow-up (25.7±0.9 mm), although in 32 patients (27.8%) mass size actually increased, while in 24 patients (20.8%) it decreased. In no case were the variations in mass dimension associated with the appearance of radiological criteria of malignancy. Kaplan–Meier curves indicated that the cumulative risk for mass enlargement (65%) and for developing endocrine abnormalities (57%) over time was progressive up to 80 months and independent of haemodynamic and humoral basal characteristics. In conclusion, mass enlargement and the presence or occurrence over time of subclinical endocrine alterations are frequent and not correlated, can appear at any time, are not associated with any basal predictor and, finally, are not necessarily indicative of malignant transformation or of progression toward overt disease.

Several algorithms have been proposed for management of patients with adrenal incidentalomas. The majority, but not all, recommend a conservative approach, except for masses suspected for malignancy and/or clearly hyperfunctioning which need surgical treatment (Jockenhovel *et al*, 1992; Ambrosi *et al*, 1997; Barzon *et al*, 1998; Barzon and Boscaro, 2000; Brunt and Moley, 2001; Thompson and Young, 2003; Mansmann *et al*, 2004). However, the follow-up of these apparently benign and nonfunctioning masses is arbitrary, reflecting the clinical experience of the individual Unit rather than deriving from clinical studies planned to evaluate this aspect. The main problem is that the natural course of adrenal incidentalomas has not yet been fully clarified. Prespecified protocols that have prospectively investigated the evolution of these masses are scanty and those reported in the literature have given inconsistent results on the basis of different duration of observation, number of patients studied and methodological approaches adopted (Jockenhovel *et al*, 1992; Terzolo *et al*, 1998; Barzon and Boscaro, 2000; Siren *et al*, 2000). In particular, it is not known if these masses may increase in size and, above all, whether this is a marker of malignant transformation or whether malignancy may also occur without size variation. In addition, the meaning of the subtle endocrine alterations frequently observed in these patients remains unclear and it is uncertain whether such abnormalities may transform into overt hypersecretion leading to classic endocrine diseases.

The present study was planned about 10 years ago in order to follow-up patients with adrenal incidentalomas after exclusion of patients with masses showing hormonal hyperfunction and/or a radiological picture of malignancy. The selected patients have been managed by morphological and hormonal investigations at regular intervals for up to 7 years.

## PATIENTS AND METHODS

### Patients

Patients with adrenal incidentalomas, that is, clinically unapparent adrenal masses incidentally detected after imaging studies for unrelated reasons (Mantero *et al*, 2000; Mansmann *et al*, 2004), were carefully selected for this study. Adopting a diagnostic procedure followed in our Unit, we submitted the patients to a basal morpho-functional study. Morphological evaluation was based on CT using a fast scanner and 3-mm scanning interval. Criteria of benignity were mass size preferably lower than 35 mm, homogeneity, regular margins, well-defined lesion, attenuation values less than 10 Hounsfield Units, with no-to-mild homogeneous CT enhancement and rapid (10–15 min) washout of more than 50% of the initial enhancement after i.v. contrast (Boland *et al*, 1998; Pena *et al*, 2000; Caoili *et al*, 2002). Hormonal evaluation included plasma ACTH and cortisol before and after 1 mg dexamethasone (DMX) suppression test, plasma renin activity, aldosterone and potassium, plasma noradrenaline and adrenaline, plasma free and total testosterone, androstenedione, dehydroepiandrosterone and 17hydroxyprogesterone. When endocrine abnormalities were found, confirmatory tests were always performed, namely 24-h urinary free cortisol and 2 and 8 mg DMX suppression tests for suspected Cushing's syndrome, captopril and saline loading tests for suspicion of primary aldosteronism, glucagon test and 24-h urinary catecholamines and metanephrines for suspected pheochromocytoma and, finally, ACTH test when 21-*β*-hydroxylase deficiency was suspected.

Using this diagnostic work-up, patients presenting radiological features of malignancy and/or hormonal hypersecretion were excluded from the study and advised to undergo surgery. Thus, only patients with radiologically benign masses and no adrenal hormonal excess, except for isolated, combined or qualitative subclinical hormone abnormalities, were admitted to a follow-up study after obtaining their written consent. The resulting group was composed of 115 patients (72 females and 43 males, mean±s.d. age 56.5±11 year, range age 16–80 year) with masses right-sided in 59 cases, left-sided in 27 cases and bilateral in 29 cases. In total, 39 patients were normotensive (systolic blood pressure 122.3±2.0 mmHg and diastolic blood pressure 76.9±1.0 mmHg, mean±s.e.) and 76 hypertensive (systolic blood pressure 156.5±1.7 mmHg and diastolic blood pressure 94.7±1.0 mmHg). Obesity or overweight was present in 43 patients (body mass index 29.3±0.5 kg m^−2^) while normal-weight was recorded in 72 patients (body mass index 23.2±0.3 kg m^−2^). In total, 14 patients suffered from diabetes while 101 patients were normoglycaemic.

The experimental design was an endocrine and morphological follow-up of at least 1 year after diagnosis in which patients were evaluated yearly for up to 7 years (median 4 year, range 1–7 year) following the same diagnostic work-up adopted in basal conditions and described above.

### Experimental design

At entry and during each follow-up, all subjects maintained their usual diet, except for diabetic patients who observed an adequate diet. Patients taking drugs (antihypertensives or other drugs) suspended medication for at least 2 weeks prior to the study. On the test day, all subjects, respecting 24-h urinary collection, underwent blood sampling for hormone measurements (between 0800 and 0900) after overnight fasting and in the sitting position. At 2300 on the same day they assumed (orally) 1 mg DXM and blood samples for cortisol determination were taken the following day at 0800. All patients then underwent CT scan with i.v. contrast.

### Assays

Hormone determinations were performed in the same laboratories throughout the years of the study, using kits from the same companies and, frequently, the same technicians. All hormones were assayed in duplicate using specific commercial RIA kits, except for catecholamines (high-performance liquid chromatography). Intra-assay and interassay CVs of the hormones were, respectively: noradrenaline 14 and 20% adrenaline 17 and 22% dehydroepiandrosterone-sulphate (Radim, Rome, Italy) 6.8 and 8.1% 17hydroxyprogesterone (ICN Biomedicals, Costa Mesa, CA, USA) 9.4 and 11.8% androstenedione (Sorin, Saluggia, Italy) 7.1 and 10.8% cortisol (Immunoteck International, Marseille, France) 5.7 and 6.6% ACTH (Nichols Institute Diagnostics, San Juan Capistrano, CA, USA) 3.1 and 7.3% aldosterone (DiaSorin, Saluggia, Italy) 9.7 and 11.5% plasma renin activity (DiaSorin, Saluggia, Italy) 7.6 and 9.1% free testosterone (Diagnostic Systems Laboratories, Inc., Webster, TX) 5.0 and 8.3% total testosterone (Medical System, Genova, Italy) 6.0 and 7.8%. Intra-assay and interassay CVs of the urinary hormones were, respectively: noradrenaline 14 and 20% adrenaline 17 and 22% normetanephrine 7.6 and 12.7% and metanephrine (Immuno Biological Laboratories, Hamburg, Germany) 18.7 and 11.9%.

### Hormonal parameters with normal values

Plasma cortisol: 60–300 ng ml^−1^; ACTH: 9–52 pg ml^−1^; aldosterone: <20 ng dl^−1^; aldosterone/plasma renin activity (ng ml^−1^ h^−1^) ratio: <70 (95% upper confidence limit of normal values obtained from 82 patients with essential hypertension); dehydroepiandrosterone-sulphate: 0.3–4.3 *μ*g ml^−1^; 17hydroxyprogesterone: 0.15–3.4 ng ml^−1^; free testosterone: <3.6 pg ml^−1^ for females and 10–40 pg ml^−1^ for males; total testosterone: 0.1–1.0 ng ml^−1^ for females and 3.0–10 ng ml^−1^ for males; androstenedione: 0.2–3.1 ng ml^−1^; noradrenaline: <600 pg ml^−1^ and adrenaline: <80 pg ml^−1^.

Urinary normetanephrine: 30–440 *μ*g/24 h; metanephrine: 20–345 *μ*g/24 h; noradrenaline <80 *μ*g/24 h; adrenaline <20 *μ*g/24 h; and dopamine <400 *μ*g/24 h.

Normal responses to captopril (50 mg os) and saline loading (2 l of saline in 4 h) tests: aldosterone values under 15 and 5 ng dl^−1^, respectively; normal response to glucagon (1 mg i.v.): plasma noradrenaline plus adrenaline <1000 pg ml^−1^; normal response to DXM: plasma cortisol <50 ng ml^−1^; normal response to ACTH (250 mg i.v.): plasma 17hydroxyprogesterone <5 ng ml^−1^.

### Radiological parameters with benign/malignant criteria

All CT scans were blinded and independently reviewed by two experienced radiologists using standardized criteria, commonly used for adrenal gland investigations (Lee *et al*, 1991; McNicholas *et al*, 1995; Korobkin *et al*, 1996; Szolar and Kammerhuber, 1997; Cirillo *et al*, 1998; Pena *et al*, 2000; Caoili *et al*, 2002; Kebapci *et al*, 2003). If discordant opinions were recorded, an additional evaluation was carried out by a third operator. The diagnosis of a benign lesion was established when the mass, well-defined, showed regular margins and homogeneity, and when attenuation values were less than 10 HU with no-to-mild homogeneous CT enhancement, and with rapid (10–15 min) wash-out of more than 50% of the initial enhancement after i.v. contrast. Other criteria of benignity, although not essential, were lack of necrosis, absence of haemorrhage or calcification and size (maximum length and maximum width) <40 mm. In the case of bilateral masses, only values of the larger mass were reported.

### Statistics

Results are given as mean±s.e. Correlations were examined by linear regression analysis. Comparisons between variables were tested with Pearson's *χ*^2^ and Student's *t*-test, as appropriate. Survival analysis was used to estimate the likelihood of developing adrenal hyperfunction, tumor enlargement or tumor reduction. Kaplan–Meier curves were generated for estimating cumulative risk of developing adrenal hyperfunction or tumor variation. To evaluate factors predictive of progressive disease, we selected and dichotomized the following parameters: sex (females *vs* males), obesity (BMI cutoff, 27 kg m^−2^), arterial hypertension (blood pressure values cutoff: 140/90 mmHg), diabetes (glycaemia value cutoff: 126 mg dl^−1^), side (monolateral *vs* bilateral, left *vs* right), endocrine abnormalities (presence or absence) and, finally, mass size (stratified in three groups: <20 mm, 20–35 mm, >35 mm).

Data were analysed using SPSS/PC+11.5 statistical software.

## RESULTS

### Hormonal picture at entry

**Figure 1:**
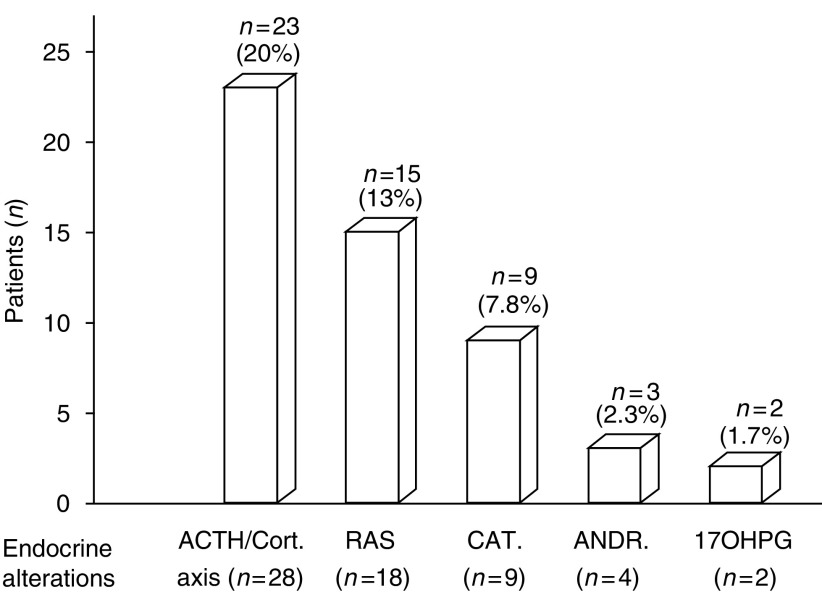
Patient distribution on the basis of endocrine alterations. ACTH/Cort=ACTH/cortisol, RAS=renin–angiotensin system, CAT=catecholamines, ANDR=androstenedione and 17OHPG=17hydroxyprogesterone.

In 62.6% of cases (72 out of 115) the endocrine pattern was normal, while in 37.4% of cases (43 out of 115) we found isolated or combined, though subclinical, endocrine alterations (Figure 1). The most frequent abnormality concerned the ACTH–cortisol axis with 28 alterations in 23 patients (*n*=6 low ACTH, *n*=1 high cortisol, *n*=7 no DXM suppression, *n*=5 low DHEA-S, *n*=1 high cortisol, no DXM suppression and low DHEA-S, *n*=1 high cortisol and no DXM suppression, *n*=2 no DXM suppression and low DHEA-S), followed by the renin–angiotensin system with 18 alterations in 15 patients (PRA suppressed in 15 patients associated with high aldosterone in three cases: all 15 were normokaliemic and 12 were hypertensive). Catecholamines presented alterations in nine patients who showed high noradrenaline levels and normal adrenaline (all nine were asymptomatic and eight had stable hypertension). Finally, we observed six alterations in androgen levels in five females (*n*=1 high testosterone, *n*=1 high androstenedione, *n*=1 both elevated, *n*=2 high 17hydroxyprogesterone) without evident clinical signs of hyperandrogenism. In summary, we observed 61 endocrine alterations in 43 patients, but in all cases confirmatory tests excluded specific endocrine diseases.

### Radiological picture at entry

Mean maximum mass diameter was 25.4±0.9 mm. In 86% of cases (99 out of 115), adrenal masses were lower than 35 mm on CT, while in the remaining 16 (14%) mass dimensions were greater. However, in all cases radiological signs of benignity were present.

### Correlations at entry

No correlation was found either between mass dimensions and endocrine alterations or haemodynamic/metabolic parameters, or between endocrine alterations and haemodynamic/metabolic parameters.

### Hormonal picture at last follow-up

Mean values of the hormonal parameters examined at the last follow-up did not significantly differ from those observed at entry (Table 1 ). However several endocrine modifications during follow-up were observed in the individual patients. Thus, 57 endocrine alterations found in 43 patients (37.2%) were no longer confirmed at follow-up, while four alterations in three patients (2.6%) persisted. In contrast, 35 new alterations in 31 patients (26.9%) appeared *de novo*. As observed in basal conditions, in these cases the hormonal abnormalities likewise concerned the ACTH–cortisol axis and, less frequently, the other hormones (Table 2 ). However, in patients in whom the endocrine alterations were confirmed or appeared *de novo* at follow-up, confirmatory tests were always negative for specific endocrine diseases.

### Radiological picture at last follow-up

On average, no variation in mass size was found between values at entry (25.4±0.9 mm) and at follow-up (25.7±0.9 mm). However, individual evaluation indicated that mass size increased in 32 patients (27.8%) and decreased in 24 (20.8%). These variations were, however, modest because in the former group the increase was ⩽5 mm in 2/3 of cases (21 out of 32 patients), while in the latter group the decrease was ⩾5 mm in only four cases. An increment greater than 10 mm was found in four cases occurring in the first 2 years without further increase at follow-up. A mass reduction greater than 10 mm was found in only two cases, probably due to spontaneous infarction and subsequent tumor involution.

Careful evaluation indicated that in no case was the variation in mass dimension associated with the appearance of radiological criteria of malignancy.

### Survival analysis

**Figure 2:**
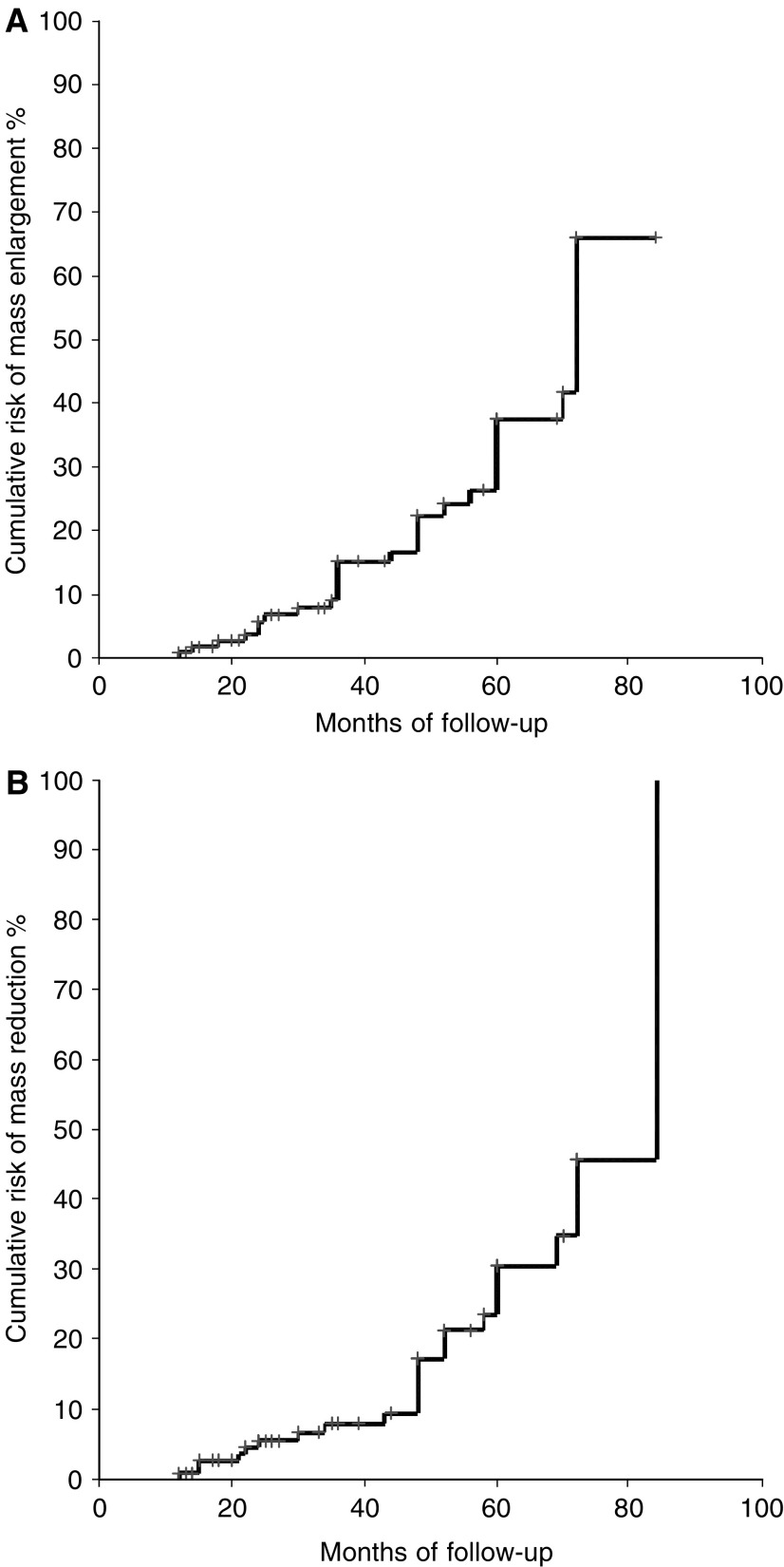
Estimated overall cumulative risk of adrenal mass enlargement (**A**) and mass reduction (**B**) over time in patients with adrenal incidentalomas (*n*=115).

Kaplan–Meier curves were used to estimate cumulative risk of developing mass variations over time. The risk of mass enlargement was globally elevated (65%) and progressive up to 80 months but similar to that for mass reduction over time (Figure 2). The risk for mass enlargement was independent of lesion side, sex and basal endocrine pattern (presence or absence of hormonal alterations), blood pressure, BMI, glycaemic pattern and mass size at entry.

**Figure 3:**
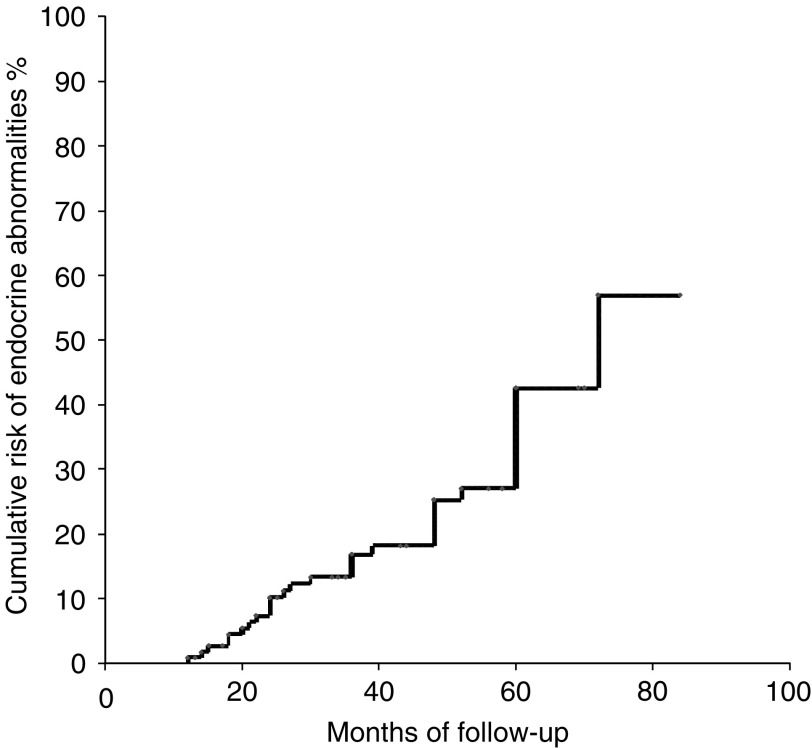
Estimated overall cumulative risk of developing endocrine abnormalities in patients with adrenal incidentalomas (*n*=115).

The estimated cumulative risk of developing endocrine abnormalities over time was globally 57% and progressive up to 80 months (Figure 3). The occurrence of endocrine abnormalities was not significantly associated with lesion side, sex and body weight, glycaemic control, blood pressure status and mass size at entry.

To date, all patients are alive except nine who died for accidental causes (*n*=2), stroke (*n*=2) or cardiovascular diseases (*n*=5). In addition, seven patients rejected further tests after the first follow-up (1 year) and 36 patients dropped out after 4 yearly observations. Seven patients developed hypertension and four diabetes during follow-up. Finally, the patients in whom hormonal abnormalities or size mass variations developed at the last follow-up were submitted to tests at least another twice, but revealed no signs of hypersecretion or malignancy of the mass.

### Clinical data in patients with adrenal mass enlargement

No difference in gender, lesion side or mass dimension (categorized in tertiles), or in presence or absence of endocrine abnormalities, obesity, diabetes and hypertension was recorded at entry among the 32 patients who subsequently developed a mass increase at follow-up. In addition, in these patients the appearance of endocrine alterations over time (nine out of 32, 28%) was similar to that observed in patients who showed a mass decrease (eight out of 24, 33.3%) and in those who presented no variation in mass dimension (18 out of 59, 30.5%).

## DISCUSSION

The management of adrenal incidentalomas is becoming an important aspect of health care, and its importance may increase further because the extensive use and advances in imaging technology could reveal an even higher incidence of adrenal masses. While several concordant diagnostic and therapeutic algorithms have been proposed for management of patients with adrenal incidentalomas, follow-up studies are scanty and those reported in the literature are not univocal, probably on account of different duration of observation, number of patients studied and methodological approaches adopted (Jockenhovel *et al*, 1992; Barzon *et al*, 1999; Siren *et al*, 2000). Barzon *et al* (1999), in the most comprehensive study to date, reported that though the risk of malignancy was very low even in the case of an increment in mass size, the large incidentalomas should be carefully followed-up due to the elevated probability of hyperfunction which, in their study, occurred in two out of 75 patients studied. Other authors have similarly reported the development of overt hypersecretion of the mass over time and even occasional malignant transformation of adrenal incidentalomas (Hensen *et al*, 1990; Jockenhovel *et al*, 1992). In contrast, no shift toward malignancy or functional hyperactivity was observed in several other studies (Osella *et al*, 1994; Barry *et al*, 1998; Terzolo *et al*, 1998; Rossi *et al*, 2000; Siren *et al*, 2000; Grossrubatscher *et al*, 2001). Thus, the natural course of adrenal incidentalomas still requires further clarification.

The novelty of the present investigation derives from several important aspects. This is a prospective study investigating the morpho-functional evolution of apparently benign and nonfunctioning adrenal masses. The study was performed on a large number of patients and the length of observation was sufficient to evaluate the possible modifications of these tumors. Furthermore, this follow-up was conducted in a single Unit with the same physicians and technicians, so that potential bias deriving from different diagnostic procedures or different laboratory methods was avoided. In addition, the majority of the patients underwent regular yearly tests and even patients who developed mass morpho-functional variations over time were submitted to tests for at least another 2 years.

Our first major finding is that, in agreement with several reports (Reincke *et al*, 1992; Fernandez-Real *et al*, 1994; Osella *et al*, 1994; Mantero *et al*, 1997; Barzon *et al*, 1998; Terzolo *et al*, 1998; Mantero *et al*, 2000; Sworczak *et al*, 2001), patients with adrenal incidentalomas frequently show isolated subtle subclinical endocrine alterations. Several terms have been adopted to define these forms, on the basis of the hormone involved, such as pre-, subclinical or silent Cushing's syndrome, and preclinical hyperaldosteronism or pheochromocytoma, but the clinical significance of these abnormalities remains unknown. In particular, we do not know whether these subclinical syndromes may result in progression towards overt diseases. On the basis of our data this does not seem to occur since in no case did confirmatory tests detect patients with specific endocrine diseases. Furthermore, these hormonal alterations normalized spontaneously over time in the majority of cases, as similarly reported by other authors (Terzolo *et al*, 1998). We also found that the risk of developing new subclinical endocrine alterations remained constant over time (even 6–7 years after the first observation) and was independent of baseline morpho-functional characteristics of the masses, indicating that neither mass size nor other parameters were predictors of these subclinical endocrine abnormalities.

The second major finding of our study concerns the morphological evolution of the mass over time. In the literature changes in size have been reported in adrenal incidentalomas (Bastounis *et al*, 1997; Barry *et al*, 1998; Terzolo *et al*, 1998; Barzon *et al*, 1999; Siren *et al*, 2000; Libe *et al*, 2002; Grumbach *et al*, 2003), with such variations sometimes believed to reflect a more aggressive growth rate (Mansmann *et al*, 2004). Barzon *et al* (1999) reported that incidentalomas tend to undergo a period of increase in mass size but thereafter remain unchanged, suggesting the possible existence of a programmed end point of growth of adrenal masses. In contrast, Grossrubatscher *et al* (2001) found that adrenal mass enlargement may occur unpredictably at any time, sometimes after a long period of ‘quiescence’. In the present study we noted frequent increments but also reductions in mass size, although such variations consistently proved to be small. In addition, the risk of mass enlargement was constant over time and no basal predictor was present either with regard to morphological characteristics, such as initial mass dimensions, or functional status, such as the presence of subclinical endocrine alterations at entry. Finally, our data showed that mass enlargement was never associated with the development of the endocrine alterations and with malignant transformation of these lesions. Thus, mass increase was casual and, in our opinion, should not be considered a marker of hormonal hyperfunction or of malignancy.

The finding that evolution of the mass towards clinically manifest or malignant forms was not observed in our study implies that follow-ups may be carried out at fairly long intervals of time. Therefore, a careful morpho-functional evaluation should be undertaken above all at the first observation of the patients, since if the mass proves to be benign and nonfunctioning this picture will probably remain unchanged in the future as well. However, the fact that size enlargement and subtle hormonal alterations may occur at any time, even after a long period of quiescence, does suggest that clinical surveillance should be maintained for a prolonged period. For these reasons, in patients with adrenal incidentalomas and no sign of hormonal hypersecretion or malignancy at the first observation we propose a follow-up involving a radiological (CT) examination every 2 years and an hormonal evaluation every 3 years up to 7–10 years. If hormonal and morphological parameters remain unchanged further follow-ups can be avoided.

In conclusion, we found that patients with adrenal incidentalomas frequently show subtle subclinical hormonal alterations not corresponding to specific endocrine diseases. In addition, we observed that mass increase and the development of new hormonal abnormalities over time are not correlated, can appear at any time, are not associated with any basal predictor and, finally, that are not necessarily indicative of malignant transformation or progression toward overt disease. Therefore, our data minimize the risk of morbidity and mortality from hormone hypersecretion or malignancy, at least for the period analyzed by our protocol, and warn of the risk of overtesting and overtreating these patients. Accordingly, a conservative approach of these masses appears reasonable, and follow-ups conducted at long intervals over a prolonged period of time seem to be the best choice for management of patients with adrenal incidentalomas.

## Figures and Tables

**Table 1:** Hormonal parameters of our patients at entry and at last follow-up

**Parameter**	**Basal (*n*=115)**	**Follow-up (*n*=115)**	***P*<**
Plasma cortisol (ng ml^−1^)	163.7±5.2	161.0±5.4	NS
Plasma cortisolo after DXM (ng ml^−1^)	25.5±2.7	21.6±1.6	NS
ACTH (pg ml^−1^)	16.2±2.0	18.0±4.1	NS
DHEA-S (*μ*g ml^−1^)	0.73±0.05	0.75±0.06	NS
Androstenedione (ng ml^−1^)	1.43±0.08	1.41±0.08	NS
Total testosterone (ng ml^−1^)	1.60±0.2	1.49±0.2	NS
Free testosterone (pg ml^−1^)	3.95±0.9	4.5±0.7	NS
17hydroxyprogesterone (ng ml^−1^)	0.90±0.06	0.85±0.06	NS
Plasma noradrenaline (pg ml^−1^)	375.4±15.8	391.9±14.6	NS
Plasma adrenaline (pg ml^−1^)	36.7±2.1	32.3±1.9	NS
Plasma Renin Activity (ng ml^−1^ h^−1^)	1.13±0.11	1.23±0.11	NS
Aldosterone (ng dl^−1^)	25.4±1.6	23.5±1.0	NS
Plasma potassium (mEq l^−1^)	4.0±0.03	4.0±0.03	NS

The results are expressed as mean±s.e.

NS=not significance.

**Table 2:** Altered endocrine picture at entry and/or at follow-up

	**Not confirmed**		**Confirmed**		***De-novo* appeared**	
**Endocrine alterations**	**Alterations no.**	**Patients no.**	**Alterations no.**	**Patients no.**	**Alterations no.**	**Patients no.**
ACTH–cortisol axis	28	23	4^a^	3	18^b^	17
Renin–angiotensin system	18	15	0	0	8^c^	5
Catecholamines	9	9	0	0	6	6
Androgens/17OH-progesterone	6	5	0	0	3^d^	3

a No suppression of DXM (two cases) and low DHEA-S levels (two cases).

b No suppression of DXM (three cases), low DHEA-S levels (10 cases), low ACTH levels (four cases), high cortisol levels (one case).

c Low PRA levels (five cases) and high aldosterone levels (three cases).

d High androstenedione levels (one case) and high 17hydroxyprogesterone levels (two cases).

The number of patients in whom hormonal alterations were not confirmed at follow-up (*n*=52) does not correspond to the number reported in the ‘Hormonal picture at last follow-up’ section (*n*=43), since some patients showed coexistence of more than one hormonal alteration.
